# Profile of calories and nutrients intake in a Brazilian multicenter study of nulliparous women

**DOI:** 10.1002/ijgo.13655

**Published:** 2021-03-24

**Authors:** Maria J. O. Miele, Renato T. Souza, Iracema M. Calderon, Francisco E. Feitosa, Débora F. Leite, Edilberto A. Rocha Filho, Janete Vettorazzi, Jussara Mayrink, Matias C. Vieira, Rodolfo C. Pacagnella, José G. Cecatti, for the Preterm SAMBA study group

**Affiliations:** ^1^ Department of Obstetrics and Gynecology School of Medicine University of Campinas (UNICAMP) Campinas Brazil; ^2^ Department of Gynecology and Obstetrics Botucatu Medical School Sao Paulo State University (Unesp) Botucatu Brazil; ^3^ MEAC—Maternity School of the Federal University of Ceará Fortaleza Brazil; ^4^ Department of Maternal and Child Health Federal University of Pernambuco Recife Brazil; ^5^ Department of Obstetrics and Gynecology Maternity Hospital Federal University of RS Porto Alegre Brazil; ^6^ Division of Women and Children's Health Faculty of Life Sciences and Medicine School of Life Course Sciences London UK

**Keywords:** energy intake, food consumption, maternal nutrition, pregnancy, ultra‐processed foods

## Abstract

**Objective:**

To assess the calorie intake and nutritional content of the maternal diet in regions with different culinary traditions and typical foods, and to understand the nutritional profile so as to provide information about the consumption of this population and promote maternal and perinatal health.

**Methods:**

From a cohort of 1145 pregnant women with diverse socio‐backgrounds we analyzed the dietary characteristics profile according to three guidelines and compared the differences between regions of Brazil.

**Results:**

Women from the northeast had the lowest level of income, occupation, education, and age (*P* < 0.001). Intakes of unprocessed/minimally processed foods and processed foods were more prevalent in women from the northeast than in southern/southeastern women (*P* < 0.001). The consumption of dairy products and vegetables was less than the recommended intake, with lower intake in southern/southeastern women (*P* < 0.001). This study showed a lower consumption of dairy and vegetables, with a shortfall of vitamins K and D, iron, calcium, folate, magnesium, and chromium from natural and fortified foods. We observed a greater consumption of unprocessed or minimally processed food in women from the northeast of Brazil.

**Conclusion:**

Our findings indicate the importance of differentiating the source of calorie intake between regional nutritional guidance and the diversity of local cuisine.

## Synopsis

Data from 1145 maternal diets show adequate intake of calories but poor consumption of dairy, vegetables, and micronutrients from natural and fortified food.

## 1 INTRODUCTION

Optimal maternal nutritional status is the result of adequate diet, body composition, and nutritional requirements during pregnancy.^1^ In the past, malnutrition was linked to famine resulting from food deprivation causing chronic energy deficiency and weight loss. A study of obstetric birth records from the time of the Dutch famine (1944–1945) found that women exposed to food deprivation during the antenatal period gave birth to smaller newborns compared with the periods before and after the Second World War.^2^ In the last decades, however, there has been a global epidemiological phenomenon referred to as “hidden hunger”, in which a diet has excess total calories, but is poor in micronutrients.^3^ A maternal diet with high quantities of calories and lower nutrients increases the risk for maternal obesity, gestational diabetes, hypertensive disorders, and large‐ or small‐for‐gestational‐age newborns.^4^ Nutrition transition is a global burden where people exchange a meal prepared with natural or minimally processed foods for fast food or "ready‐to‐eat, ready‐to‐heat" meals that belong to the ultra‐processed food groups, which are rich in calories and poor in nutrients.^5^ Nowadays, malnutrition coexists with obesity, causing non‐communicable diseases related to diet, and increasing the need for evaluating and recommending healthier and more sustainable diets.^6^

The present study aimed to discover the dietary habits of pregnant women in different regions of the country using a broad approach to caloric density, which is a new method to evaluate food consumption involving three guidelines for the caloric assessment of foods and the ability to supply vitamins and minerals. Data were obtained from different contexts and food patterns in Brazil. In the Brazilian northeast, there is a hot and humid climate and there a variety of fresh fruits, cassava, couscous, coconut, beans, and nuts from Pará are widely used in the traditional gastronomy. In the south and southeast regions, the climate is milder and people generally consume rice and beans, potatoes, pasta, and more red meat and chicken.^7^ With the variety of nutritional patterns in a country as diverse as Brazil, our aim was to assess the calorie intake and the nutritional content of the maternal diet according to region. With each region's culinary traditions and typical foods, we aimed to understand the nutritional profiles so as to provide information about the consumption of these populations and to contribute to promoting maternal and perinatal health.

## 2 MATERIALS AND METHODS

This study is an analysis of secondary objectives from the multicenter prospective cohort study "Preterm SAMBA—Preterm Screening And Metabolomics in Brazil and Auckland",^8^ involving a comprehensive cohort with nulliparous women from any economic and educational status without serious comorbidities at 19–21 weeks of pregnancy when entering the study. The study was held between July 2015 and July 2018 in five public tertiary hospitals for specialized obstetric care in different geographical regions of Brazil: Recife and Fortaleza in the northeast, with a mean lower income; Porto Alegre in the south; and Campinas and Botucatu in the southeast, with the highest mean incomes in the country.

**FIGURE 1:**
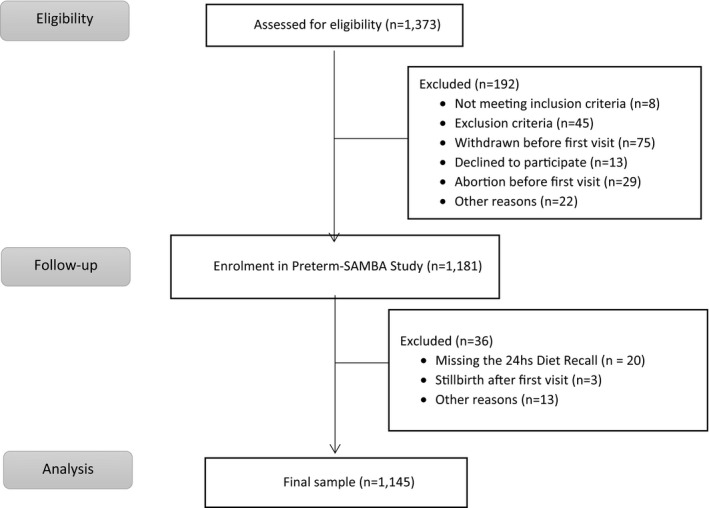
Flowcharts of eligible participants from analysis and final sample

The included criteria were women who were pregnant for the first time or nulliparous women, with a singleton pregnancy, who responded to the 24‐h diet recall interview and who were between 19 and 21 weeks pregnant (Fig. 1). Gestational age was confirmed by an early ultrasonography. Exclusion criteria were any risks associated with preterm birth, provision of special diet counseling, maternal chronic illness, and a reproductive history with more than two previous abortions. Women were also not included in the study in cases of cervical suture; fetal malformation; chronic hypertension requiring antihypertensive drugs; diabetes or renal disease as self‐reported by the women themselves; arterial blood pressure equal to or above 160/100 mm Hg on enrollment; systemic lupus erythematosus or antiphospholipid syndrome; sickle cell disease; HIV infection; Müllerian anomalies; history of cervical knife cone biopsy; chronic use of corticosteroids, aspirin, calcium, fish oil, vitamin C, vitamin E or heparin. All centers used nutritional assessment tools (anthropometry and 24‐h recall dietary intake), on the first visit. Diagnostic criteria for hypertension in pregnancy and diabetes mellitus followed international guidelines.^9^, ^10^ Data on newborn weight, length and head circumference were evaluated according to the World Health Organization guidelines and placenta weight was also collected.^11^ Sociodemographic characteristics were self‐reported. All data were inserted in an electronic platform (medscinet AB).

This study aimed to assess the mean dietary values of the meal preparation and serving size details from two population groups, using a single day of intake recorded per person through one 24‐h diet recall, which, according to Willett,^12^ is adequate for the profile of this study. Interviews were performed by trained local staff using the multi‐step method.^13^ We estimated serving sizes based on a photographic album of household measures. To standardize servings, household measures were converted into grams or milliliters of consumption using Brazilian reference manuals.^14^ The type and amount of meal intakes were listed for breakfast, lunch, snacks (morning and afternoon) and dinner.

The nutrients selected for assessment were evaluated using the estimated average requirements (EAR) or adequate intake, and were adopted according to dietary reference intakes (DRIs) for pregnant women.^15^ To analyze these specific nutrients, food composition, data from Brazilian tables, and the National Nutrient Database for Standard Reference of the US Department of Agriculture were used.^16^, ^17^ We also extracted information from industrial food labels and cooking recipes. dietwin^®^plus software (version 3090) was used for all data collection and dietary analyses.

Energy was calculated from the amount of foods and was divided into groups defined by their characteristics of food sources and degrees of processing. The total diet and the calories consumed were therefore organized and analyzed according to the criteria of three guidelines, respectively: the DRIs, the NOVA classification, and the Brazilian Food Pyramid.^15^, ^18^, ^19^ We assessed total energy intake from the DRIs and compared their adequacy with standard recommendations. Calorie quality by the degree of processing of food was assessed according to the NOVA Food classification. The characteristic of calories in each predominant food group was detailed using the Food Pyramid. To calculate the estimated energy requirement of basal metabolism in calories, the predictive equation indicated in the DRIs was used. Data collection of the NOVA food regimens followed procedures according to the three food groups in the classification: ultra‐processed (UPF), processed (PF) and unprocessed or minimally processed (UMPF) foods. We added fats, salt, and sugars into culinary preparation, computed as UMPF. Calories and grams for each selected group were then calculated.

From the total sample, pregnant women enrolled were allocated into two groups: northeast (NE) and south/southeast (S/SE) of Brazil, according to the region in which they lived. Descriptive statistics were calculated and presented as number (percentage), mean (standard deviation), or median. To compare socioeconomic and maternal outcome differences between regions, the *χ* ^2^ test was applied for categorical variables. For the nutrients of maternal diet, data variables were analyzed using Mann–Whitney tests to compare the maternal differences between regions, and the normality tests Kolmogorov–Smirnov, Shapiro–Wilk and Anderson–Darling. All results with a *P* value below 0.05 were considered significant. The software/program R 3.6.1 was used (R Foundation for Statistical Computing, Vienna, Austria, 2019. URL http://www.R‐project.org) to filter variables, cleaning and mining data, calculations and statistics, and the graphics were created using graphpad prism version 8.3.0 (GraphPad Software, La Jolla, CA, USA).

All women signed an individual two‐way informed consent form before admission to the study. The Preterm‐SAMBA Study followed the terms of the Helsinki Declaration (2013). It was approved by the institutional review boards of all participating centers (coordinating center protocol 20182318.8.0000.5404), in addition to the National Ethics Committee for Research (CONEP).

## 3 RESULTS

From the total of women included in the Preterm‐SAMBA study, a total of 1145 women met criteria for this analysis according to the flowchart in Figure 1.

Table 1 shows descriptive characteristics of the study population according to two regions. Women from the S/SE use proportionally less public prenatal care, had a higher income, spent more years in school, and had a higher rate of paid work, when compared with NE women. The greatest sociodemographic differences were between maternal skin color/ethnicity, source of prenatal care, and income. Despite the difference in income and education between regions (*P* ≤ 0.001), the Human Development Index was shown to be insignificant (*P* = 0.067). Regarding maternal anthropometric measurements, women from the S/SE had greater weight, height, lean body mass (skinfolds), and head circumference. However, women from the NE had higher measurements related to muscle mass (muscle arm circumference and arm muscle area bone‐free). S/SE newborns had smaller length and cephalic perimeters than infants from the NE. The NE group had more pre‐eclampsia, whereas women from the S/SE had a higher incidence of gestational diabetes mellitus.

**TABLE 1:** Distribution of maternal sociodemographic features by Brazilian region^a^

Maternal features^b^	Southeast/south	Northeast	*P* value^k^
Income^c^ (US$ per year)			<0.001
<3000	5 (0.8)	42 (7.6)	
3000–6000	48 (8.1)	200 (36.0)	
>6000–12 000	169 (28.7)	206 (37.1)	
>12 000	367 (62.3)	108 (19.4)	
Occupation			<0.001
Paid work	363 (61.6)	216 (38.8)	
Housewife	93 (15.8)	115 (20.7)	
Not working^k^	133 (22.6)	225 (40.5)	
Marital status			0.063
With partner	440 (74.7)	388 (69.7)	
Without partner	149 (25.2)	168 (30.2)	
Maternal skin color/ethnicity^c^			<0.001
White	352 (60.8)	90 (16.2)	
Black	61 (10.5)	53 (9.6)	
Brown	162 (28.0)	407 (73.5)	
Other	4 (0.7)	4 (0.7)	
Maternal age, year			<0.001
<20	122 (20.7)	162 (29.1)	
20–34	410 (69.6)	369 (66.3)	
>34	57 (9.6)	25 (4.4)	
Education, year			<0.001
<9	45 (7.6)	124 (22.3)	
9–12	331 (56.1)	310 (55.7)	
>12	213 (36.1)	122 (21.9)	
Source of prenatal care^c^			<0.001
Entirely public	453 (78.2)	524 (94.6)	
Private/insurance/mixed	126 (21.8)	30 (5.4)	
Human Development Index	0.763	0.803	0.067
Maternal anthropometry^l^			
Weight, kg	69.6 ± 15.3	66.2 ± 13.5	<0.001
Height^d^, m	1.62 ± 0.1	1.59 ± 0.1	<0.001
BMI^e^	26.5 ± 5.6	26.1 ± 4.9	0.713
Obese^c^ (BMI >30.9)	108 (18)	88 (15)	0.253
Non‐obese (BMI ≤30.9)	481 (81)	467 (84)	0.517
^c^Skinfolds^l^, mm			
Triceps	22.3 ± 7.9	20.9 ± 7.2	0.004
Biceps	15.4 ± 7.1	15.0 ± 6.7	0.316
Subscapular	22.1 ± 9.7	20.6 ± 7.7	0.068
Suprailiac	23.3 ± 9.4	19.1 ± 8.2	<0.001
Sum of four skinfolds	83.3 ± 30.5	75.7 ± 26.2	0.001
Circumferences^l^, cm			
Head^f^	55.3 ± 1.9	54.8 ± 1.8	<0.001
MUAC^g^	28.7 ± 4.6	28.5 ± 4.6	0.888
MAC^g^	21.7 ± 3.1	22.1 ± 3.4	0.027
AMA^h^	32.0 ± 11.3	33.4 ± 12.3	0.027
Maternal outcomes			
Placenta^i^, g	569.5 ± 139.7 (*n* = 325)	544.8 ± 174.9 (*n* = 54)	0.208
Hypertensive disorders^j^	31 (5.4)	53 (9.6)	0.006
SGA^j^	70 (12.1)	73 (13.2)	0.558
Preterm birth^j^	66 (11.4)	56 (10.1)	0.352
Gestational diabetes^c^	60 (10.7)	37 (6.7)	0.015
Newborn outcomes			
Birth weight, kg	3107 ± 572.8	3154 ± 619.1	0.086
Cephalic perimeter	33.9 ± 1.8	34.1 ± 2.0	0.029
Length	48.0 ± 3.0	48.3 ± 3.3	0.030
Total	589	556	

Abbreviations: AMA, arm muscle area bone‐free; BMI, body mass index (calculated as weight in kilograms divided by the square of height in meters);MAC, muscle arm circumference; MUAC, mid upper arm circumference; SGA, small for gestational age.

^a^ Values are given as number (percentage) or as mean ± standard deviation.

^b^ Missing information indicated as follows: ^c^ *n* = 12; ^d^ *n* = 1; ^e^ *n* = 34; ^f^ *n* = 36; ^g^ *n* = 27; ^h^ *n* = 29; ^i^ *n* = 766; ^j^ *n* = 13.

^k^ *P* value from *χ* ^2^ or Mann–Whitney *U* test.

^l^ Measured during the first study visit (19–21 weeks of pregnancy).

**FIGURE 2:**
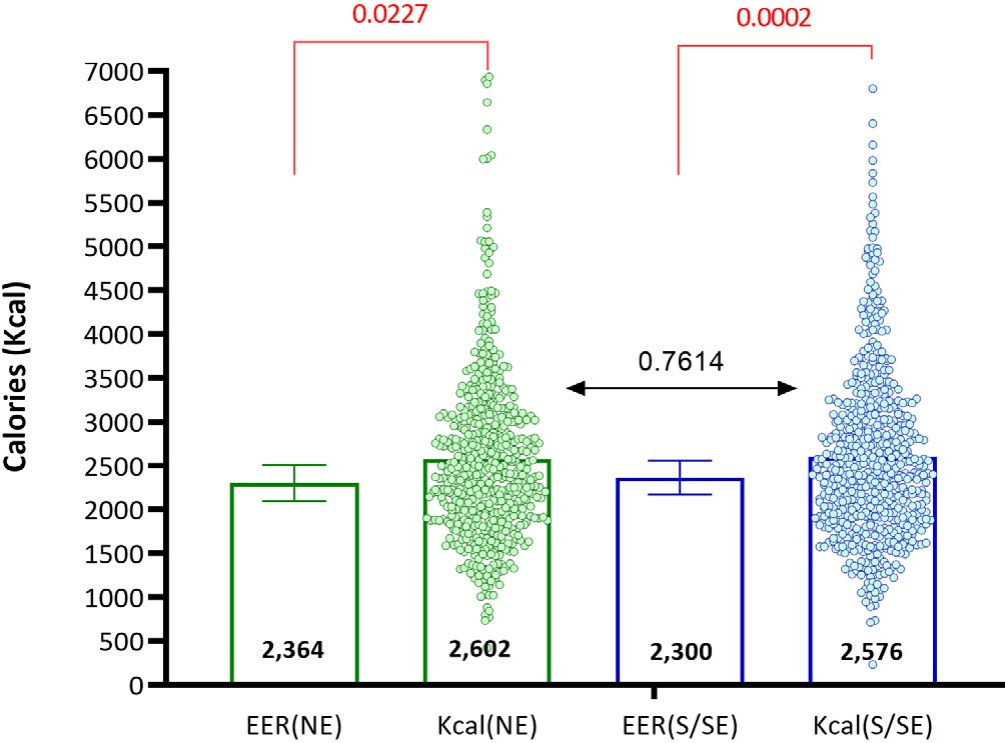
Calorie intake and estimated energy requirement (EER) per region in Brazil. *P* value: Mann–Whitney *t*‐tests. Estimated energy requirement was calculated according to dietary reference intakes, considering maternal age (<19 and ≥19 years). Colored dots represent the EER recommendation, according to each region and calorie consumption per pregnant woman. Kcal, caloric intake per woman according to the region of the country

Figure 2 depicts the results for caloric intake in both groups, as well as caloric intake obtained from the 24‐h diet recall calculated according to DRI (estimated energy requirement) recommendations. The percentages of the adequacy of macronutrients in relation to guidelines were: carbohydrate caloric intake was 51.88% in S/SE women and 50.7% in NE women; lipid caloric intake was 38.94% in S/SE women and 26.8% in NE women; and protein intake was 16.6% in S/SE women and 16.6% in NE women. The results in comparison with the DRI recommendations showed that the percentage fat intake as a percentage of energy in the NE was above recommended values (15%–30%). Both regions had a greater protein intake (10%–15%) and lower carbohydrate intake (55%–75%) than indicated by the DRIs. Although these differences were not significant, macronutrients contributed to the largest portions of calorie intake. There was a high consumption of fatty acids, 36.2% of which were saturated fatty acids in NE women, compared with 30.5% (*P* < 0.001) in S/SE women. The percentage of monounsaturated fatty acids was 29.4% in the NE group and 25.6% in the S/SE group (*P* = 0.003). Most dietary saturated fatty acids were derived from animal products.

**FIGURE 3:**
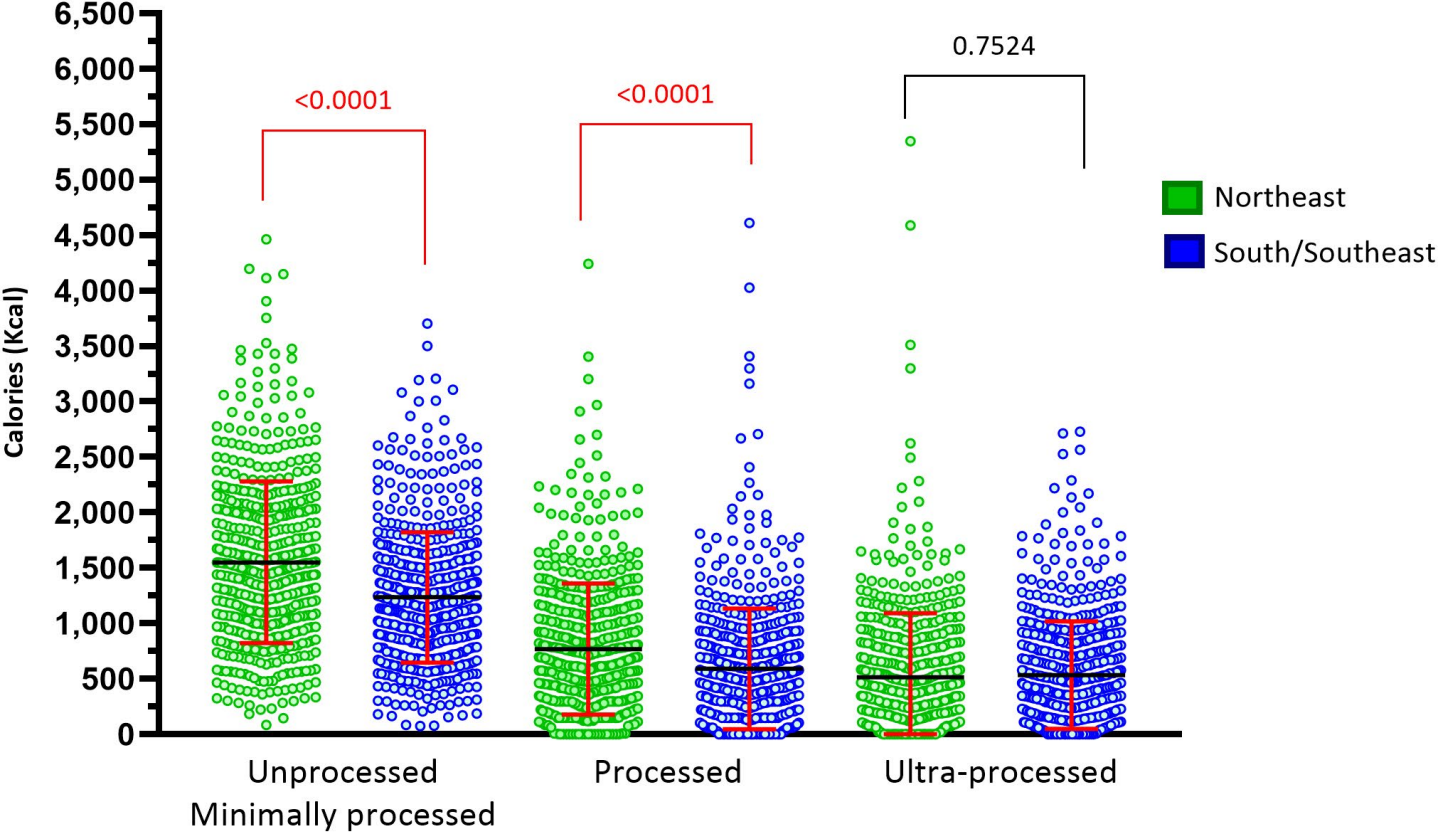
The distribution of calorie intake according to the degree of food processing (NOVA Food) by regions of Brazil. The *P* value shows average caloric intake between groups according to the degree of food processing (Mann–Whitney *U* tests). The colored points represent intake per woman

Calorie quality is shown in Figure 3, according to the three degrees of food processing (NOVA Food). From the total calories in S/SE women, 52.3% originated from UMPF, 25.0% from PF, and 22.6% from UPF. In contrast, for NE women, 54.7% were derived from UMPF, 27.1% from PF, and 18.2% for UPF. These differences between groups were significant (*P* < 0.05). The selection by NE women of more UMPF and less UPF reflected the eating habits of the population, and highlighted the difference in calorie intake between regions. This apparently small 2% difference in UMPF consumption is clinically relevant in the long‐term, as these foods contain more fibers, bioactive agents and vitamins.

In Table 2, calories are arranged per regions by food characteristics according to the Food Pyramid groups. The intake of dairy products and vegetables was lower than guideline recommendations for both regions, influencing micronutrient intake. Total calorie intake was assessed and resulted mainly from grains (949.9 kcal), meat/eggs (628.4 kcal), and fat. Nevertheless, we observed that NE women reported selecting their major servings from home‐cooked meals of local cuisine.

**TABLE 2:** Daily servings of different food categories and their adequacy in the Food Pyramid (Brazilian guideline) according to regions of Brazil (*n* = 1145)^a^

Food group adequacy^b^	Southeast/south (*n* = 589)	Northeast (*n* = 556)	*P* value^c^
Grains	87.9%	116.8%	<0.001
	5.1 (0.2, 32.3)	6.6 (0.4, 34.6)	
	5.6 ± 3.3	7.5 ± 4.4	
Vegetables	63.2%	88.3%	0.398
	1.6 (0.0, 94.4)	1.7 (0.0, 67.1)	
	2.8 ± 5.5	3.9 ± 6.9	
Fruits	159.8%	150.2%	0.762
	4.3 (0.1, 33.0)	4.4 (0.1, 28.2)	
	5.9 ± 5.1	5.6 ± 4.4	
Dairy products	59.7%	63.5%	<0.001
	1.5 (0.1, 12.8)	1.5 (0.2, 8.4)	
	1.8 ± 1.2	1.9 ± 1.4	
Meat/eggs	209.6%	278.8%	0.874
	2.3 (0.1, 16.6)	3.1 (0.2, 16.6)	
	2.7 ± 1.9	3.7 ± 1.9	
Beans	272.7%	412.2%	<0.001
	2.6 (0.0, 25.6)	3.7 (0.0, 23.6)	
	3.6 ± 3.1	5.6 ± 4.7	
Sweets	176.5%	153.1%	0.449
	1.5 (0.1, 18.1)	1.6 (0.1, 11.8)	
	2.4 ± 2.3	2.0 ± 1.8	
Fat	379.3%	396.4%	0.683
	3.4 (0.0, 37.4)	3.0 (0.0, 59.6)	
	5.1 ± 5.0	5.2 ± 5.1	

^a^ Values are given as percentages, median (minimum, maximum), or as mean ± standard deviation.

^b^ The percentage of adequacy with the Pyramid food portions are derived from mean values according to the minimum and maximum intake for each food group. For each food group one serving portion corresponded to: Grains, 150 kcal, Vegetables, 15 kcal, Fruits, 35 kcal, Dairy products, 120 kcal, Meat/eggs, 190 kcal, Beans, 55 kcal, Sweets, 110 kcal, Fat, 73 kcal.

^c^ *P* values are from the Mann–Whitney *U* test.

Table 3 shows the difference in vitamin and mineral intake between regions, and the percentage of adequacy in DRI reports according to median intake. Staple foods and fortified foods were analyzed. We accessed vitamin K1 (menaquinones) and K2 (phylloquinone) values separately. From the total intake, we found mean values of 3.4 and 11.6 µg, and median values of 0.6 and 0.7 µg, for the NE and S/SE regions, respectively. Although none of the reported intakes met EAR levels for chromium, levels in NE women were substantially below the EAR. NE women were more likely to choose foods that were poor sources of chromium, compared with S/SE women. This difference between regions resulted in only 10% of the food sources for NE women.

**TABLE 3:** Maternal vitamin and mineral intake and their adequacy in the estimated average requirement or adequate intake (dietary reference intakes) according to region of Brazil (*n* = 1145)^a^

Adequacy^b^	Southeast/south (*n* = 589)	Northeast (*n* = 556)	*P* value^c^
Vitamin B12 (µg)	185.0%	183.0%	0.681
	4.1 (0.0, 164.5)	4.0 (0.0, 509.0)	
	6.3 ± 14.9	12.7 ± 45.3	
Vitamin C (mg)	130.0%	103.8%	0.207
	91.0 (0.0, 1907.0)	72.7 (0.0, 1977.0)	
	134.9 ± 179.3	160.9 ± 225.5	
Vitamin D (IU)	30.9%	50.9%	<0.001
	3.1 (0.0, 132.8)	5.1 (0.0, 40.7)	
	4.8 ± 8.9	6.6 ± 5.9	
Folate (µg)	36.6%	35.0%	0.826
	190.4 (18.1, 1586.0)	182.2 (3.7, 1885.0)	
	217.3 ± 142.9	244.7 ± 209.3	
Vitamin K (µg)	4.1%	2.6%	<0.001
	3.7 (0.0, 542.2)	2.3 (0.0, 873.5)	
	20.6 ± 58.1	9.3 ± 39.8	
Calcium (mg)	73.0%	77.3%	0.347
	584.1 (64.1, 5618.0)	618.3 (9.8, 3322.0)	
	679.3 ± 466.8	693 ± 430	
Iron (mg)	54.0%	58.0%	0.886
	11.9 (2.3, 84.4)	12.8 (0.7, 80.8)	
	14.6 ± 9.5	14.3 ± 8.8	
Zinc (mg)	104.8%	105.5%	0.558
	11.5 (1.5, 55.7)	11.6 (1.2, 194.9)	
	13.4 ± 8.3	13.0 ± 10.6	
Chromium (µg)	34.8%	3.3%	<0.001
	10.5 (0.0, 264.1)	1.0 (0.0, 254.0)	
	29.7 ± 41.1	12.9 ± 30.4	
Magnesium (µg)	83.8%	95.4%	<0.001
	251.4 (1.4, 737.0)	286.3 (3.67, 754.1)	
	269.2 ± 112.5	305.1 ± 133.2	
Selenium (µg)	100.2%	158.2%	<0.001
	49.1 (0.0, 361.9)	77.5 (0.0, 697.4)	
	60.8 ± 52.3	91.8 ± 73.2	

^a^ Values are given as percentages, median (minimum, maximum), or as mean ± standard deviation.

^b^ The percentage of adequacy in the dietary reference intakes is median nutrient intake according to maternal age for: Calcium =800 mg (>18 years).

^c^ *P* values were obtained using Mann–Whitney *U* tests.

## 4 DISCUSSION

This study confirmed that the adequacy of energy intake cannot reflect the quality of the nutritional content, with a deficit of >60% of the vitamins and minerals assessed. Moreover, poor consumption of vegetables and dairy is a worrying habit for pregnant women, as these are important sources of nutrient targets during this period of life. On the other hand, the wide difference of UMPF and PF intake between regions has demonstrated the influence of diet patterns over the food choices.

Traditional meals made with mostly fresh foods have been switched to UPF, fast‐food, high in calories, but with fewer vitamins and minerals. In this study, it seems to affect the upper classes more than the lower classes, which can be explained because industrial foods are more expensive than fresh foods in Brazil. In contrast, UPF represents the major percentage of energy intake in high‐income countries, with 57.9% in the USA, and 47.7% in Canada. In middle‐income countries, fast‐food consumption is lower (29.8% in Mexico, 28.6% in Chile, and 21.5% in Brazil).^20^ However, in this study, eating more UMPF by the NE women did not ensure adequate micronutrient consumption for all. The poor intake of chromium by the two groups with wide differences between regions may be explained by the eating habits in NE, where women are more likely to replace bread and wheat flour with cassava and couscous, which have little or no chromium. Although chromium is ubiquitous in food, at very low concentrations, most of it is derived from food processing using stainless steel equipment, so diets composed of unprocessed foods contain significantly less chromium than diets that have undergone processing.^21^ The consumption of these micronutrients during pregnancy is associated with maternal glycemia. Magnesium functions as a cofactor for enzymes of carbohydrate metabolism that can modulate insulin resistance.^22^ Meals of NE women were mostly composed of cassava, vegetables, traditional dishes made with beans, dairy products, and nuts. Northeastern food patterns have a lower glycemic index compared with meals composed of white bread with ham and cheese, fruit juice, coffee with sugar, pizza, and pasta, which are seen more frequently in the S/SE meal pattern, Nevertheless, both groups were shown to have unhealthy outcomes, with higher GDM values in the S/SE, and pre‐eclampsia in the NE.

Moreover, none of the groups reached the recommendation for the consumption of dairy products and vegetables, and the levels of calcium were low, and very low for vitamin K. Dairy products and dark green leafy vegetables are a source of calcium, folate, vitamin K, and iron. Milk has the highest bioavailability of calcium, while dark green leafy vegetables have the highest bioavailability of vitamin K1. The bioavailability of calcium depends on physiological and dietary factors including consumption of food sources, pregnancy, lactation, and vitamin D intake. Fresh dark leafy greens with vegetable oils provide a bioavailable source of vitamin K1, which has an important function for prothrombin in blood coagulation.^23^

Along with the lower levels of vitamin K, the present study found that vitamin D food sources were below the recommendation of DRIs for pregnancy. Vitamin D and calcium at low levels or if a pregnant woman consumes vitamin K antagonists, can induce soft‐tissue calcification and increase the risk for atheroma formation. Indications of high doses of vitamin D should be used with caution.^24^

In summary, our results showed that the eating habits of the regions of Brazil had widely been influenced by the same pattern for the deficiencies or excess of vitamins and minerals of food sources. We observed poor consumption of dairy and vegetables, insufficient intake of vitamins K and D, and of minerals iron, calcium, folate, magnesium, and chromium from both natural and fortified foods. However, women in the NE region appear to be less affected by the food processing industry compared with women in the S/SE. We suggest that dietary patterns should not be evaluated in isolation, but according to the sources of calories, and the characteristics of regional cuisines, to avoid the lack of nutrients. This study outlined a profile of maternal nutrition from different cultures in Brazil; however, new studies could evaluate the best interventionist approach to improve nutritional status in pregnancy.

## CONFLICTS OF INTEREST

The authors have no conflicts of interest. The lead author affirms that this manuscript is an honest, accurate, and transparent account of the study being reported. The reporting of this work is compliant with STROBE guidelines. The lead author affirms that no important aspects of the study have been omitted.

## AUTHOR CONTRIBUTION

MJM, RTS, JM, RCP, MCV, and JGC designed the study; MJM, RTS, JM, IMC, FEF, DFL, EAR, and JV conducted data collection; MJM, JGC, and MCV conducted data analysis. All authors had access to the data and participated in the interpretation of results. MJM wrote the first draft of the manuscript, reviewed initially by JGC and then by all authors who read, revised, and approved the final version submitted for publication.
